# Isl2b regulates anterior second heart field development in zebrafish

**DOI:** 10.1038/srep41043

**Published:** 2017-01-20

**Authors:** Hagen R. Witzel, Sirisha Cheedipudi, Rui Gao, Didier Y. R. Stainier, Gergana D. Dobreva

**Affiliations:** 1 Origin of Cardiac Cell Lineages Group, Max Planck Institute for Heart and Lung Research, Bad Nauheim, Germany; 2 Department of Developmental Genetics, Max Planck Institute for Heart and Lung Research, Bad Nauheim, Germany; 3 Medical Faculty, University of Frankfurt, 60590 Frankfurt am Main, Germany

## Abstract

After initial formation, the heart tube grows by addition of second heart field progenitor cells to its poles. The transcription factor Isl1 is expressed in the entire second heart field in mouse, and *Isl1*-deficient mouse embryos show defects in arterial and venous pole development. The expression of Isl1 is conserved in zebrafish cardiac progenitors; however, Isl1 is required for cardiomyocyte differentiation only at the venous pole. Here we show that Isl1 homologues are expressed in specific patterns in the developing zebrafish heart and play distinct roles during cardiac morphogenesis. In zebrafish, *isl2a* mutants show defects in cardiac looping, whereas *isl2b* is required for arterial pole development. Moreover, *Isl2b* controls the expression of key cardiac transcription factors including *mef2ca, mef2cb, hand2* and *tbx20*. The specific roles of individual Islet family members in the development of distinct regions of the zebrafish heart renders this system particularly well-suited for dissecting Islet-dependent gene regulatory networks controlling the behavior and function of second heart field progenitors in distinct steps of cardiac development.

The heart is generated by distinct progenitor cell populations, which have specific regional contributions to the developing heart. The earliest population of cardiac progenitors, the first heart field (FHF), fuses at the midline and differentiate into the myocardium of the heart tube. After formation of the initial heart tube, the heart grows by the addition of second heart field (SHF) progenitor cells to its arterial and venous poles^1^,^2^,^3^. These progenitor cells, located in the splanchnic mesoderm, are patterned along the anterior-posterior axis into anterior and posterior SHF adjacent to the arterial and venous poles of the heart, respectively^4^,^5^. The anterior SHF contributes to the formation of right ventricular and outflow tract myocardium^6^,^7^, whereas the posterior SHF contributes to atrial myocardium and the sinoatrial node^8^. Defects in the SHF are largely responsible for the high rate of congenital cardiac abnormalities in humans, underscoring the importance of understanding the molecular mechanisms regulating SHF-mediated cardiogenesis^9^. Isl1, the best-established SHF marker in mouse, is transiently expressed in SHF progenitors before migration into the heart tube and is downregulated during their differentiation^1^,^10^. Isl1-deficient mouse embryos lack the outflow tract, right ventricle, and a large portion of the atria, all structures derived from the SHF, as Isl1 is required for the proliferation, survival, and migration of these cells into the forming heart^1^,^11^. Moreover, Isl1 is instrumental for cardiac progenitor cell function by coordinating genome organization and transcriptional regulation upstream of a gene regulatory network driving cardiac differentiation and SHF development^12^,^13^. However, dissection of the regulatory networks downstream of Isl1 in the anterior and posterior SHF that control the formation of distinct regions of the heart has been difficult as Isl1 is expressed in both domains of the SHF.

Despite morphological differences between the zebrafish and the mammalian heart, the zebrafish has emerged as a powerful model system to study heart development and cardiovascular disease, due to the increasing evidence for genetic conservation between zebrafish and mammalian cardiogenesis^14^,^15^,^16^. However, it has so far remained unclear if the role of Isl1 proteins is also conserved in the zebrafish system. Isl1 is expressed in zebrafish SHF progenitors^17^,^18^, but, in contrast to mammals, Isl1 is required for cardiomyocyte differentiation only at the venous pole of the heart^19^. We therefore set out to investigate whether additional Islet family members are required for the development of the arterial pole of the heart.

## Results and Discussion

### Islet family members are expressed in distinct patterns in the developing zebrafish heart

**Figure 1:**
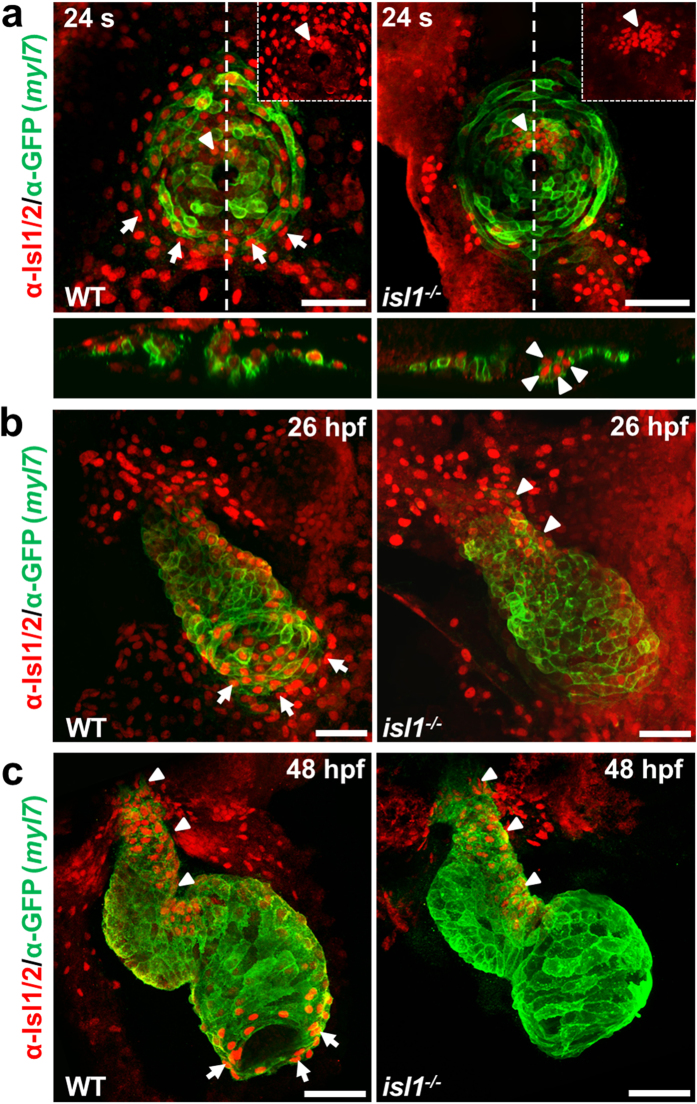
Residual Isl1/2 positive cells in *isl1*−/− zebrafish hearts. (**a**–**c**) Confocal images of wild-type sibling and *Tg(myl7:EGFP-HsHRAS*)^*s883*^ *isl1*−/− embryos stained with anti-GFP and anti-Isl1/2 antibodies at 24 somites (**a**), 26 hpf (**b**) and 48 hpf (**c**). Arrows point to Isl1^+^ cells at the periphery of the cone (**a**) or Isl1^+^ cardiomyocytes at the venous pole of the atrium (**b**,**c**), arrowheads point to residual Isl1/2^+^ cardiomyocytes in the future ventricle (**a**) or the inner curvature of the ventricle and the outflow pole (**b**,**c**). Scale bars, 50 μm.

**Figure 2:**
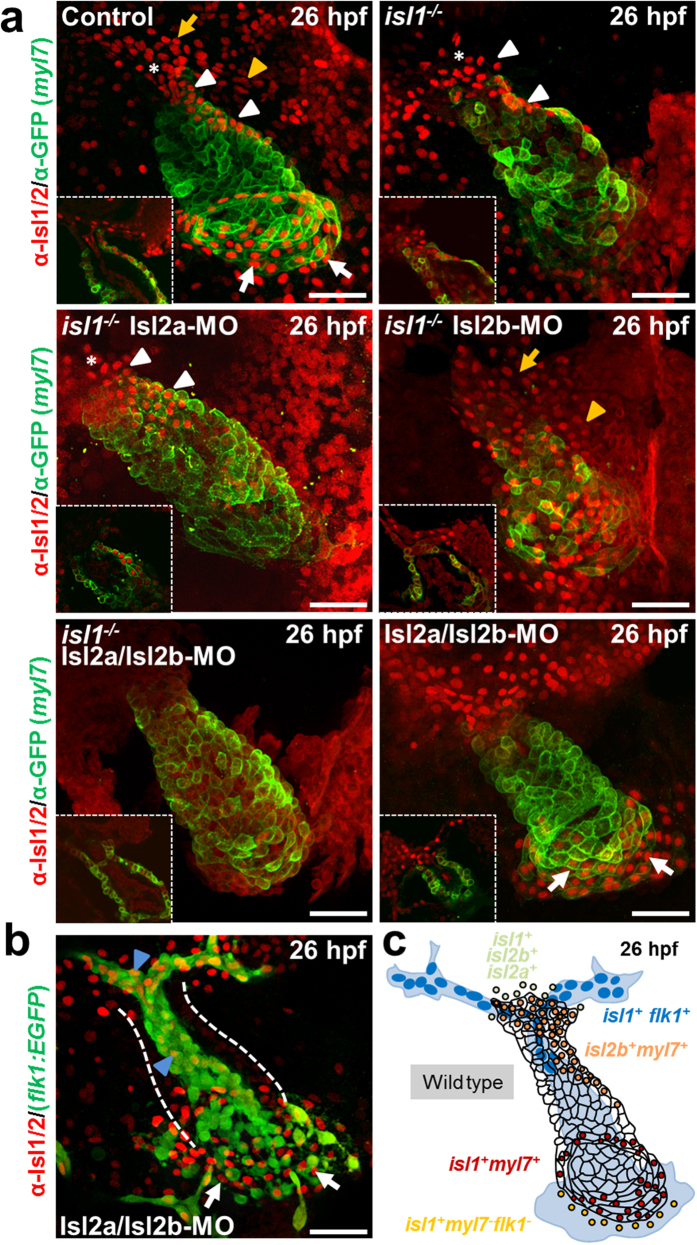
Islet family members are expressed in distinct patterns in the developing heart. (**a**) Confocal images of control and *Tg(myl7:EGFP-HsHRAS*)^*s883*^*isl1*−/− embryos or *Tg(myl7:EGFP-HsHRAS*)^*s883*^ *isl1*−/− embryos following morpholino-mediated knockdown of *Isl2a, Isl2b* or *Isl2a*/*Isl2b* stained with anti-GFP and anti-Isl1/2 antibodies at 26 hpf. (**b**) Confocal images of *Tg(flk1:EGFP) Isl2a/Isl2b* morpholino-injected embryo stained with anti-GFP and anti-Isl1/2 antibodies at 26 hpf. Asterisk indicates the late ventricular region^22^. White arrows indicate Isl1^+^ cardiomyocytes at the venous pole of the atrium; yellow arrows point to Isl2a^+^ cells in the pericardial wall; yellow arrowheads point to Isl2a^+^ cells in the adjacent endoderm; white arrowheads point to Isl2b^+^ cardiomyocytes at the inner curvature of the ventricle and the outflow pole; blue arrowheads point to Isl1^+^ endothelial cells. Scale bars in (**a**,**b**), 50 μm. (**c**) Schematic representation of the distinct Isl1^+^, Isl2a^+^ and Isl2b^+^ populations at the linear heart tube stage.

To characterize whether other Islet family members may play a role in arterial pole development during zebrafish cardiogenesis, we performed immunostaining of *Tg(myl7:EGFP-HsHRAS*)^*s883*^ embryos, that expresses membrane-bound GFP (mGFP) under the control of the *myl7* (cardiac myosin light chain 2) promoter in all differentiated cardiomyocytes, and *isl1*−/− *Tg(myl7:EGFP-HsHRAS*)^*s883*^ embryos, using an antibody recognizing both Isl1 and Isl2 proteins^18^,^20^,^21^ (Fig. 1). At cardiac cone stage (24 somites) *isl1*-deficiency led to a loss of Isl1/2 signal in the cells at the periphery of the cone, which will form the future atrium. Interestingly, residual Isl1/2-positive cells were found in cardiomyocytes of the future ventricle (Fig. 1a). Similarly, we observed residual Isl1/2-positive cells at the inner curvature of the early ventricle, the outflow pole and in the late ventricular region^22^ of *isl1* mutant embryos at 26 hours post-fertilization (hpf), whereas the Isl1/2 staining signal was completely lost at the venous pole (Fig. 1b). At 48 hpf no Isl1/2 staining was detected at the venous pole and the atrium was shorter in *isl1*−/− embryos, consistent with a previous report, showing that *isl1* is required to complete cardiomyocyte differentiation at the venous pole^19^. However, we still detected residual Isl1/2-positive cells at the arterial pole of the heart and the inner curvature of the ventricle (Fig. 1c). In zebrafish there are four annotated *Islet* family members (*isl1, isl2a, isl2b* and *isl1l*). Isl1, Isl2a, Isl2b show high overall homology (>70% identity) to each other, whereas Isl1l shares significantly less similarity (Supplementary Fig. 1a–c). All four family members share the same domain organization: two N-terminal LIM domains and one C-terminal DNA-binding homeodomain (Supplementary Fig. 1c). To analyze whether the *isl1* homologues might also play a role during cardiogenesis, we first performed qPCR analysis for *isl1, isl2a, isl2b* and *isl1l* at 10 somites, 26 hpf, 30 hpf and 48 hpf. *Isl1, isl2a, isl2b* were highly expressed at all developmental stages analyzed, whereas *isl1l* expression was not detected (Supplementary Fig. 1d). Therefore, we concentrated our further studies on *isl2a* and *isl2b*. Furthermore, Isl2a and Isl2b show high overall identity to mouse and zebrafish Isl1 (Supplementary Fig. 1e) and are recognized by the anti-Isl1/2 antibody (Supplementary Fig. 1f), suggesting that they might be expressed in the residual Isl1/2-positive cells detected in the *isl1* mutant heart, and might play a role in arterial pole development. In order to characterize the expression pattern of Islet family members during heart development in more detail, we performed *in situ* hybridization for *isl2a* and *isl2b*, together with immunostaining for Isl1/2 proteins, following morpholino mediated knockdown of *isl2a*^21^ and/or *isl2b* in *isl1*−/− embryos at 26 hpf (Fig. 2, Supplementary Fig. 1g–j). *In situ* hybridization revealed that *isl2b* is expressed in the developing heart tube, whereas *isl2a* appeared not to be expressed there (Supplementary Fig. 1g). Detailed localization studies using Isl1/2 immunostaining of MO-mediated *isl2a* knockdown *isl1*−/− embryos revealed residual Isl1/2-positive cells at the inner curvature of the early ventricle and the outflow pole (Fig. 2a). Knockdown of Isl2b in *isl1*−/− embryos led to a loss of Isl1/2-expressing cells at the inner curvature of the early ventricle, whereas residual Isl1/2-positive cells were detected in the pericardial wall and the adjacent endoderm (Fig. 2a, Supplementary Fig. 2). Depletion of all Islet family members caused a complete loss of Isl1/2-positive cells (Fig. 2a). Double knock-down of both *isl2a* and *isl2b* in control *Tg(myl7:EGFP-HsHRAS*)^*s883*^ embryos led to a complete loss of Isl1/2-positive cells at the inner curvature of the early ventricle, whereas the Isl1/2-positive cells at the venous pole of the atrium were not affected. The residual Isl1/2-positive cells in *Isl2a*/*Isl2b*-deficient embryos at the arterial pole were Isl1+/Flk1+ endothelial cells (Fig. 2b, Supplementary Fig. 2b). Thus, our data show that Islet family members are expressed in distinct patterns in the developing heart (Fig. 2c). *Isl1* is expressed in the endocardium of the forming ventricle, in the vessels at the arterial pole as well as in cardiomyocytes at the venous pole of the heart and cells at the periphery of venous pole. *Isl2a* is expressed in the pericardial wall and the adjacent endoderm and *isl2b* at the inner curvature of the early ventricle and at the periphery of the arterial pole (Fig. 2c).

### Isl2b regulates anterior second heart field development in zebrafish

**Figure 3:**
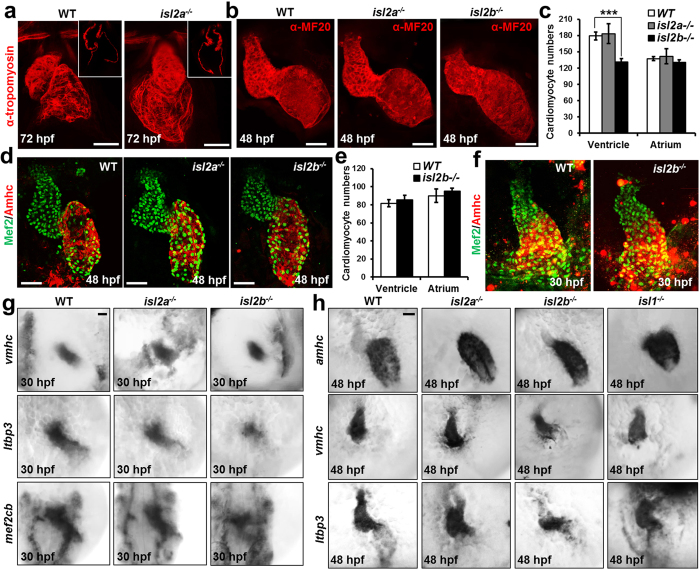
Isl2b-deficiency leads to defects in anterior SHF development. (**a**) Confocal images of control and *isl2a*−/− hearts stained with anti-tropomyosin antibody at 72 hpf, showing impaired displacement of the ventricle towards the right side. (**b**) Confocal images of control, *isl2a*−/− and *isl2b*−/− embryos stained with anti-MF20 antibody at 48 hpf. *Isl2a*−/− hearts were imaged from the side to analyze the role of *isl2a* in heart chamber formation. Scale bars in (**a**,**b**), 50 μm. (**c**,**d**) Number of atrial and ventricular cardiomyocytes (**c**) quantified following whole mount immunostaining with anti-Mef2 and anti-Amhc antibody (S46) of wild-type, *isl2a*−/− and *isl2b*−/− embryos at 48 hpf (**d**). (**e**,**f**) Number of atrial and ventricular cardiomyocytes (**e**) quantified following whole mount immunostaining with anti-Mef2 and anti-Amhc antibody (S46) of wild-type and *isl2b*−/− embryos at 30 hpf (**f**). (**g**) *In situ* hybridization for *vmhc, ltbp3* and *mef2cb* of control, *isl2a*−/− and *isl2b*−/− embryos at 30 hpf. (**h**) *In situ* hybridization for *vmhc, amhc* and *ltbp3* of control, *isl2a*−/−, *isl2b*−/− and isl1−/− embryos at 48 hpf. Data information: In (**c**), data are presented as mean ± SEM. ***p < 0.001 (Student’s t-test).

The expression pattern of *isl2a* and *isl2b* suggest that they might play a role in anterior SHF development (Fig. 2a,b). To dissect the function of isl2a and isl2b in zebrafish cardiogenesis, we generated *isl2a*−/− and *isl2b*−/− mutant zebrafish using the Transcription Activator-Like Effector Nuclease (TALEN)-mediated gene editing technology^23^,^24^ (Supplementary Fig. 3). Heterozygous carriers of either mutant allele show no obvious phenotype. Importantly, homozygous mutants for either *isl2a* or *isl2b* display cardiac abnormalities. *Isl2a*−/− embryos show defects in the displacement of the ventricle towards the right side, placing the ventricle dorsal to the atrium at 72 hpf (Fig. 3a). At 48 hpf the relative position of both chambers to each other appears to be unaffected. Chamber formation and ballooning also appear to be normal (Fig. 3b). The embryos survive for the first week, but no adult mutant fish could be found. In contrast *isl2b*−/− embryos show a significantly smaller ventricle and pronounced cardiac looping defects at 48 hpf (Fig. 3b). The ventricle and atrium remain medial and linear and acquire a “string-like morphology” later during development (at 72 hpf) possibly due to an increasing pericardial edema. The blood flow finally stops after the heart tube has completely collapsed. *Isl2b*−/− embryos do not survive the first week of development. Importantly, the number of ventricular cardiomyocytes was significantly decreased in *isl2b*−/− embryos at 48 hpf, but not at linear heart tube stage (Fig. 3c–f). The number of atrial cardiomyocytes at both stages was not changed (Fig. 3c–f). In contrast, the numbers of both atrial and ventricular cardiomyocytes were not changed in *isl2a*−/− mutants (Fig. 3c,d). Taken together, these data suggest that Isl2b is required for anterior SHF development in zebrafish. To confirm this hypothesis, we performed *in situ* hybridizations for *mef2cb* and *ltbp3*, which have been shown to identify the SHF in zebrafish^22^,^25^, and *vmhc* at 30 hpf and 48 hpf. At the linear heart tube stage we did not observe significant differences in the vmhc expression domain of *isl2a*−/− and *isl2b*−/− mutant zebrafish embryos compared to wild-type embryos. However, we found dramatic downregulation of *mef2cb* and *ltbp3* in *isl2b*−/− embryos (Fig. 3g). In contrast, *isl2a*−/− mutants did not show obvious differences in *mef2cb* and *ltbp3* expression (Fig. 3g). At 48 hpf, the *vmhc* expression domain was significantly shorter in *isl2b*−/− mutants, whereas the *amhc* expression domain was shorter in *isl1*−/− embryos, consistent with a previous report^19^ (Fig. 3h). Additionally, we observed dramatic downregulation of *ltbp3* at the arterial pole of the heart in *isl2b*−/− embryos, but no change in *isl2a*−/− and *isl1*−/− mutants (Fig. 3h). Taken together, these data show that *isl2b* is required for anterior SHF development in zebrafish and regulates myocardial addition to the arterial pole.

### Isl2b controls the expression of key regulators of cardiogenesis

**Figure 4:**
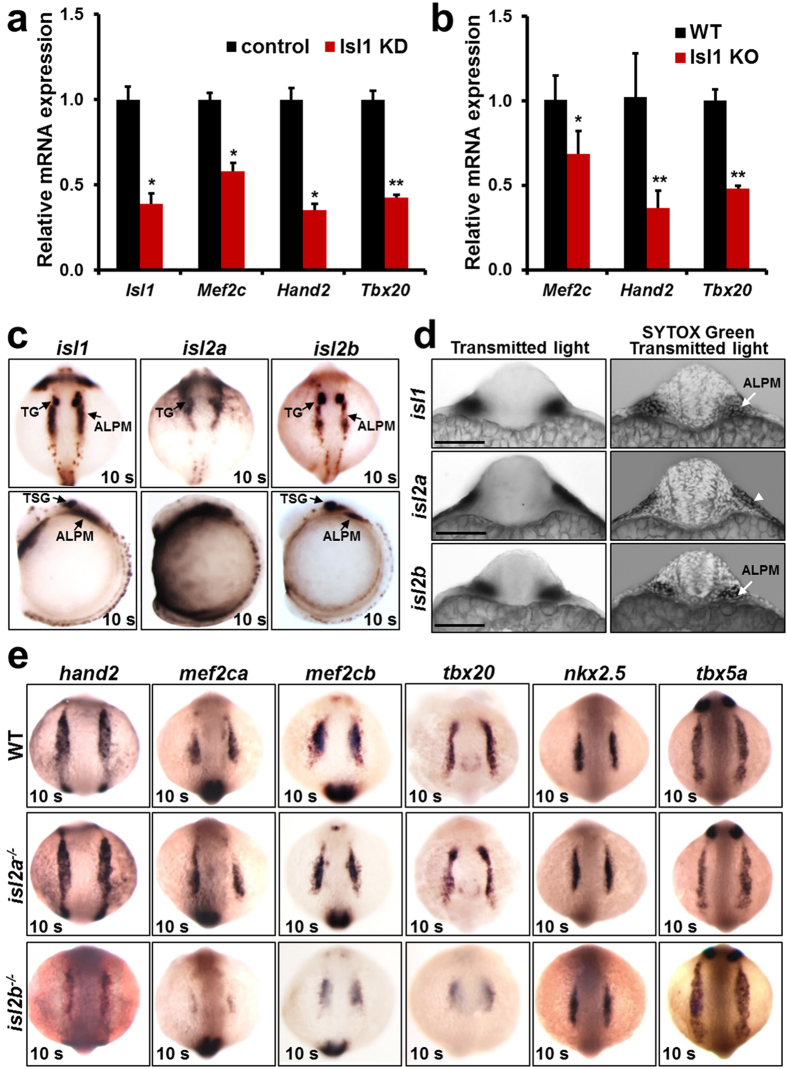
Isl2b controls the expression of key regulators of cardiogenesis. (**a**,**b**) Relative mRNA expression of the Isl1 direct targets, *Mef2c, Hand2* and *Tbx20* in control and Isl1 knockdown mouse ES cells-derived embryoid bodies after 5 days of differentiation, a stage enriched in cardiac progenitors (**a**) and in dissected SHF of E9.25 Isl1 knockout mouse embryos (**b**). (**c**) *In situ* hybridization for *isl1, isl2a* and *isl2b* expression in zebrafish embryos at the 10 somite stage. (**d**) Transverse sections after *in situ* hybridization for *isl1, isl2a* and *isl2b* (**c**) counterstained with Sytox Green and imaged with a confocal microscope. Arrows point to *isl1* and *isl2b* expressing cells in the cardiogenic region of the ALPM and in the TG (trigeminal placodes). *Isl2a* expression is observed in the periderm (arrowhead). Scale bars, 100 μm. (**e**) *In situ* hybridization for *hand2, mef2ca, mef2cb, tbx20, nkx2.5* and *tbx5a* expression in control, *isl2a*−/− and *isl2b*−/− zebrafish embryos at the 10 somite stage. Data information: In (**a**,**b**), data are presented as mean ± SEM. *p ≤ 0.05, **p ≤ 0.01 (Student’s t-test).

In mouse Isl1 plays an instrumental role in SHF cardiac progenitors and binds to key regulators of anterior SHF development, such as *Mef2c, Hand2* and *Tbx20*^12^,^13^,^26^. The expression of these genes is strongly downregulated in embryoid bodies derived from mouse embryonic stem cells expressing shRNA against *Isl1* and differentiated for 5 days, a stage enriched in cardiac progenitors (Fig. 4a), as well as in dissected SHF of *Isl1* knockout mouse embryos (Fig. 4b). To test whether Isl2b in zebrafish, similarly to Isl1 in mouse, regulates the expression of these key regulators of cardiogenesis in cardiac progenitors, we first analyzed the expression pattern of *Islet* family members in zebrafish embryos at early developmental stages by *in situ* hybridization. At 10 somites, similarly to Isl1^18^, Isl2b positive cells are found in bilateral populations of cells lying within the anterior lateral plate mesoderm (ALPM), a region known to contain heart precursor cells^14^, whereas Isl2a is expressed in the epidermal ectoderm (periderm) but not in the ALPM (Fig. 4c,d, Supplementary Fig. 4). Next, we performed *in situ* hybridization for key regulators of cardiogenesis, including *hand2, mef2ca, mef2cb, tbx20* in wild-type, *isl2a*−/− and *isl2b*−/− embryos at 10 somites. The expression of nkx2.5 and tbx5a was unchanged in both *isl2a*−/− and *isl2b*−/− embryos. In contrast, similar to the results in mouse, the expression of *hand2, mef2ca, mef2cb* and *tbx20* was strongly downregulated in *isl2b*−/− zebrafish embryos, whereas their expression appeared unaffected in *isl2a* mutants (Fig. 4e). We noticed that isl2b expression only partly overlapped with the expression domain of *hand2, mef2ca, mef2cb* and *tbx20* at 10 somites. At earlier developmental stages *isl2b* is broadly expressed in the ALPM (Supplementary Fig. 4), which may account for the downregulation of these genes in a more broader domain than those of *isl2b* at 10 somites. However, we cannot exclude the possibility that Isl2b might also indirectly regulate the expression of these genes by influencing other cell populations. Thus, *isl2b* controls the expression of *hand2, mef2ca, mef2cb* and *tbx20*, key transcription factors required for heart development.

Although the zebrafish heart consists of only a single atrium and a single ventricle, there is increasing evidence for genetic conservation between zebrafish and mammalian heart development. The discovery of a conserved SHF in zebrafish, and transcription factors (*tbx1, mef2cb, nkx2.5, hand2*) and signaling molecules (*fgf8, bmp*, RA, tgf-β) with conserved function during SHF development, further strengthen the evidence for conservation between zebrafish and mammalian cardiogenesis^15^,^17^,^22^,^25^,^27^,^28^,^29^. In mouse, the SHF is patterned into anterior and posterior domains localized next to the arterial and venous poles of the heart, respectively^3^,^5^. Isl1, the principal SHF marker in mice, plays a key role in both populations, and Isl1-deficient mouse embryos show abnormalities at both the arterial and venous poles^1^. We and others have reported that in zebrafish Isl1/2-positive cells are found adjacent to both the arterial and the venous pole, similarly to the mouse^17^,^18^,^19^. However, in contrast to *Isl1*−/− mouse embryos, *isl1*−/− zebrafish embryos show defects in cardiomyocyte differentiation only at the venous pole, leading to significantly shortened atria but a normal ventricle^19^. Our data show that Islet family members are expressed in discrete patterns in the developing heart and play a conserved role in controlling all aspects of SHF development between zebrafish and mammals. However in contrast to mouse embryos, where only one Islet family member, Isl1, is required for the development of both the arterial and venous poles of the heart, in zebrafish Isl2b control the development of the arterial pole (anterior SHF development) and Isl1 the development of the venous pole (posterior SHF). This is consistent with our previous findings that loss of function of a negative regulator of Isl1 proteins leads to significantly increased numbers of cardiomyocytes at both the arterial and the venous pole^18^.

As mutations in ISL1 in humans lead to congenital heart defects^30^, elucidating the molecular mechanisms upstream and downstream of Isl1 proteins will contribute to better understanding of cardiogenesis and will provide insights into the causes of congenital heart defects and potential therapies for them. The conserved role of Islet family members in zebrafish heart development establishes the utility of zebrafish as a powerful model organism to study these mechanisms and to dissect the gene regulatory networks controlling the behavior and function of anterior and posterior SHF progenitor cells in the formation of distinct regions of the heart in unparalleled detail.

## Methods

### Zebrafish strains

All animal experiments were done in accordance to the institutional guidelines and are covered in an approved animal experimental protocol by the Committee for Animal Rights Protection of the State of Hessen (Regierungspraesidium Darmstadt, Germany, Experimental protocol Az.: V54 – 19 c 20/15 – B2/1043). Embryos and adult zebrafish were raised under standard laboratory conditions at 28 °C. The following mutant and transgenic lines were used: *Tg(myl7:EGFP-HsHRAS*)^*s883 *^18, *Tg(-5.1myl7:nDsRed2*)^*f2*^, *Tg(kdrl:EGFP*)^*s843 *^31 and isl1sa0029 (Sanger Institute, Zebrafish Mutation Resource).

### Morpholino-mediated knockdown

For knockdown of *isl2a* and *isl2b*, embryos were injected with a total of 3.67ng morpholino (Gene Tools): *isl2a* trMO: 5′-GGATGCGGTAGAATATCCACCATAC-3′ and/or *isl2b* spMO: 5′-GTGTAAATACCTACTTTTGGAATGA-3′.

### Establishment of *isl2a* and *isl2b* mutant lines

TALEN constructs targeting exon 2 of *isl2a* and *isl2b* were generated as described previously^24^. 100 pg of 5′-capped mRNA encoding the left and right TALEN arms were injected into one-cell stage embryos. These mosaic embryos were raised to adulthood and out-crossed with WT fish in order to identify F0 founders. F1 heterozygous fish, which carried a 10 bp deletion mutation in the targeted site for *isl2a* and a 2 bp deletion for *isl2b* (Supplementary Fig. 3), were selected and out-crossed with WT fish. The F2 heterozygous progeny were inter-crossed to generate homozygous *isl2a*−/− and *isl2b*−/− embryos. Genotyping was conducted as follows: genomic DNA was extracted from 1-2 dpf embryos or a clipped tail fin of adult fish and amplified by PCR using the following primers: 5′-gtcggctgtggaagtcagat-3′ and 5′-attctgcgcacttcagacag-3′ for *isl2a*, and 5′-cagtcagatccacgaccagt-3′ and 5′-tggttgcactccacacattt-3′ for *isl2b* followed by High Resolution Melt Analysis (HRMA) analysis and sequencing.

### *In situ* hybridization, whole mount immunostaining and confocal microscopy

*In situ* hybridization and whole-mount staining were performed as described in ref. 18. Briefly, for whole-mount immunostaining embryos were fixed with 2% formaldehyde in 0.1 M PIPES (Sigma), 1 mM MgSO_4_ and 2 mM EGTA overnight at 4 °C (pH of the solution 7.4). Embryos were washed with PBS and blocked for 1 h in PBS with 5% BSA (Albumin fraction V, Sigma) and 0.3% Triton X-100 at 37 °C. The embryos were then incubated with primary antibodies diluted in blocking solution (α-Isl1/2 supernatant 1:10 (DSHB, 39.4D5); α-GFP 1:500 (Novus Biologicals, NB600-308); α-MEF-2 (C-21) 1:250 (Santa Cruz, sc-313); α-MHC supernatant 1:10 (DSHB, MF20); α-MYH6 supernatant 1:10 (DSHB, S46)) overnight at 4 °C. Embryos were washed three times with 0.3% Triton X-100 in PBS for 1 h at 4 °C and incubated with secondary antibodies diluted in blocking solution (Alexa conjugates 1:500) overnight at 4 °C. Final washes were done with 0.3% Triton X-100 in PBS, three times for 1 h at 4 °C. For sectioning, embryos were embedded in 17% gelatin in PBS. The gelatin cubes were fixed overnight at room temperature in 4% PFA in PBS and sections were performed with a Vibratome VT 1000 S (Leica). Confocal images were acquired on a Zeiss LSM 710 system and Z-stacks projections were generated using Zeiss LSM 710 software.

### Embryonic stem cell growth and differentiation

Mouse embryonic stem (ES) cells were grown on mitomycin treated mouse embryonic fibroblasts in the presence of 4.5 mg/ml D-glucose (GIBCO), containing 15% serum along with 2 mM L-Glutamine (GIBCO), 0.1 mM 2-mercaptoethanol (Sigma), 1 mM sodium pyruvate (Invitrogen) in the presence of 1,000 U/ml of leukemia inhibitory factor (LIF ESGRO, Millipore ESG1107). ES cells were differentiated into embryoid bodies (EBs) using the hanging drop method^32^. Briefly 33,000 cells/ml were aggregated by the hanging drop method and after 2 days the resulting EBs was transferred to bacterial dishes and grown for 3 days to obtain EBs enriched in cardiac progenitors.

### Generation of stable ES cell lines

0.5 × 10^6^ HEK293T cells were transfected with 2 μg of shRNA against *Isl1* (CGGCAATCAAATTCACGACCA) and Control (pLKO) plasmids (Sigma; TRC shRNA library) along with packaging and envelope plasmids using FuGENE (Roche) transfection reagent. The viral supernatant was collected 48 h after transfection and used to transduce ES cells. 24 h after transduction ES cells were selected with 10 μg/ml puromycin (BD Bioscience) for two passages.

### Mouse lines and analysis

Isl1Cre/+mice^33^ were inter-crossed to generate homozygous Isl1 knockout embryos. Staged E9.25 embryos were used to dissect cardiogenic mesoderm^12^. Tissue was resuspended in TRIzol (Invitrogen) and RNA isolation was carried out using manufacturer’s instructions.

### Q-PCR analysis

cDNA synthesis was performed using High-Capacity cDNA Reverse Transcription Kit and random primers (Applied Biosystems). Q-PCR was performed using SYBR green (Applied Biosystems) on StepOnePlus™ Real-Time PCR System (Applied Biosystems). *Gapdh* was used to normalize the gene expression changes in mouse ES cells and embryos, whereas *EF1alpha* was used in zebrafish. Relative gene expression changes were calculated using the 2^−ΔΔCt^ method. The relative mRNA abundance was calculated using the 2^−ΔCt^ method. Primers sequences are listed in Supplementary Table 1.

## Additional Information

**How to cite this article**: Witzel, H. R. *et al*. Isl2b regulates anterior second heart field development in zebrafish. *Sci. Rep.* **7**, 41043; doi: 10.1038/srep41043 (2017).

**Publisher's note:** Springer Nature remains neutral with regard to jurisdictional claims in published maps and institutional affiliations.

## Supplementary Material

Supplementary Information
