# Supplementary material for: Isl2b regulates anterior second heart field development in zebrafish
Source: Sci Rep. 2017 Jan 20;7:41043. doi: 10.1038/srep41043 (PMC5247716; doi:10.1038/srep41043)
Supplement: Supplementary Information [file srep41043-s1.pdf]

Supplementary Information

Isl2b regulates anterior second heart field development in zebrafish

Hagen R. Witzel[1], Sirisha Cheedipudi[1], Rui Gao[1], Didier Y.R. Stainier[2] and Gergana D. Dobreva[1,3*]

[1] Origin of Cardiac Cell Lineages Group, Max Planck Institute for Heart and Lung Research, Bad Nauheim, Germany

[2] Department of Developmental Genetics, Max Planck Institute for Heart and Lung Research, Bad Nauheim, Germany

[3] Medical Faculty, University of Frankfurt, 60590 Frankfurt am Main, Germany

* Correspondence: Gergana.Dobreva@mpi-bn.mpg.de

Prof. Dr. Gergana Dobreva, Goethe University Frankfurt and Max Planck Institute for Heart and Lung Research, Parkstr. 1, 61231 Bad Nauheim, Germany

Tel. +49-06032-705-259

Fax: +49-06032-705-211

Email: Gergana.Dobreva@mpi-bn.mpg.de

Supplementary Information

Supplementary Data

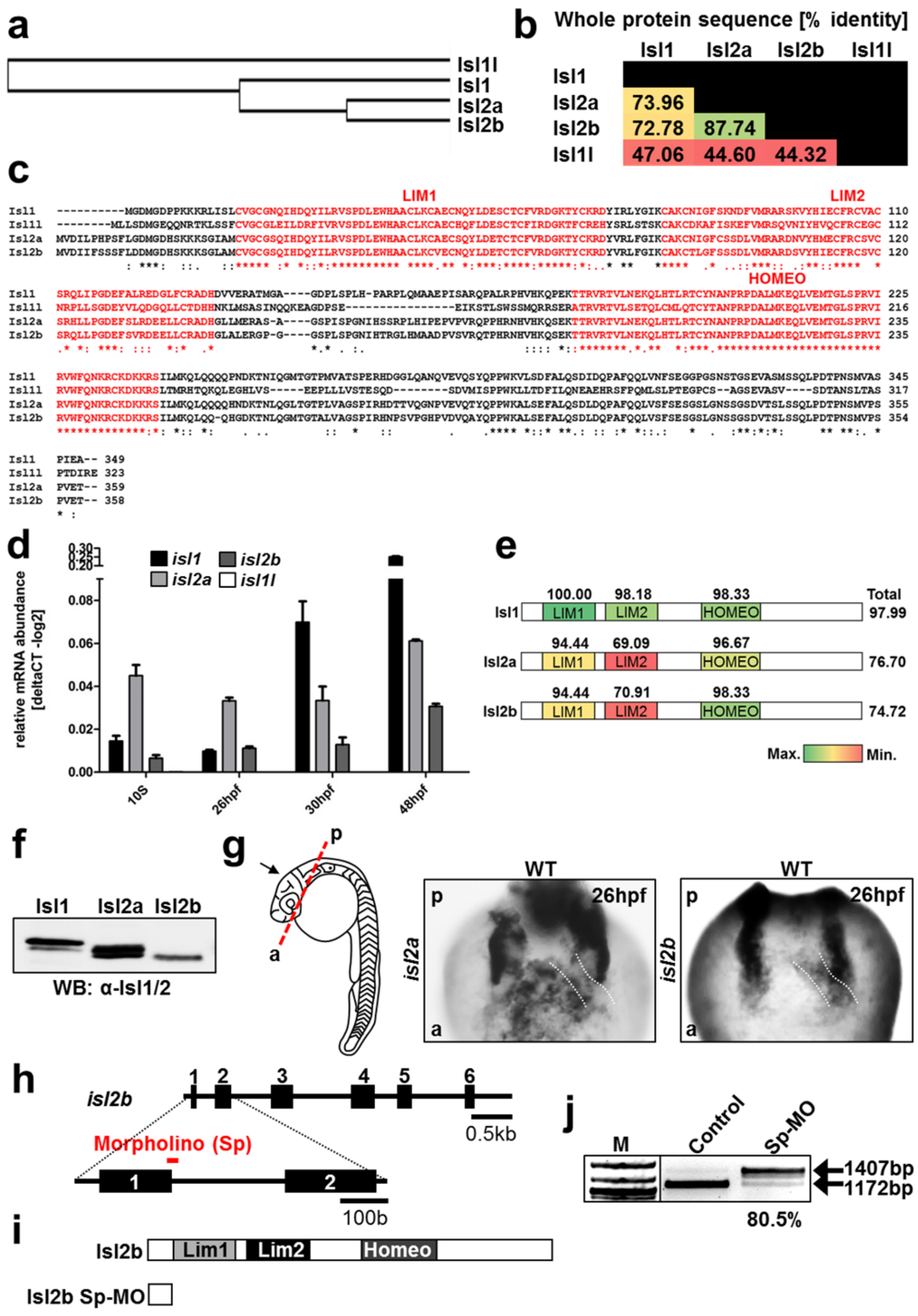

Supplementary Figure 1: Conservation and expression of Islet family members.

(a) Guide tree of Isl1, Isl1l, Isl2a and Isl2b protein alignment (*D. rerio*) generated using the Protein Knowledgebase (UniProtKB). **(b)** Amino acid sequence alignment of Isl1, Isl1l, Isl2a and Isl2b (*D. rerio*) performed with Blastp (NCBI). Values represent % of identical amino acids. Red color indicates a lower percentage and green color a higher percentage of identity. **(c)** Amino acid sequence alignment of Isl1, Isl1l, Isl2a and Isl2b (*D. rerio*) performed using the Protein Knowledgebase (UniProtKB). The LIM1, LIM2 domains and the homeodomain (HOMEO) are highlighted in red. Asterisks (*) indicate positions with a single, fully conserved residue. Colons (:) indicate conservation of amino acids with highly similar properties and periods (.) with weakly similar properties. **(d)** Relative mRNA expression of *isl1*, *isl1l*, *isl2a* and *isl2b* at 10 s, 26 hpf, 30 hpf and 48 hpf. **(e)** Schematic representation of Isl1, Isl2a and Isl2b (*D. rerio*). The functional domains, as well as the entire amino acid sequences were aligned to mouse Isl1, whose protein sequence is identical to that of human Isl1. Numbers represent the percentage of identity of zebrafish Isl1, Isl2a and Isl2b with mouse Isl1. **(f)** Western blot analysis of total protein extracts from HEK293T cells transiently expressing zebrafish *isl1*, *isl2a* and *isl2b* using an anti-Isl1/2 antibody. **(g)** *In situ* hybridization for *isl2a* and *isl2b* at 26 hpf. Before *in situ* hybridization, the head was removed as indicated by the red dashed line and images were taken in the direction of the black arrow. White dashed lines indicate the position of the heart tube. The highly stained bilateral structures represent the pharyngeal arches. **(h)** Schematic representation of the *isl2b* genomic locus and the binding sites of the morpholino oligo (shown in red). The loss of proper exon-intron junction recognition leads to an altered mRNA containing a premature stop-codon. **(i)** Schematic representation of the Isl2b protein and Isl2b-morphant protein (bottom). **(j)** RT-PCR analysis of *isl2b*-splice morpholino-injected embryos. The efficiency of *isl2b* Sp-MO was analyzed by PCR using primers spanning the first intron of the *isl2b* gene. This analysis indicated loss of spliced *isl2b* mRNA (1172 bp band) in embryos injected with the *isl2b* Sp-MO.

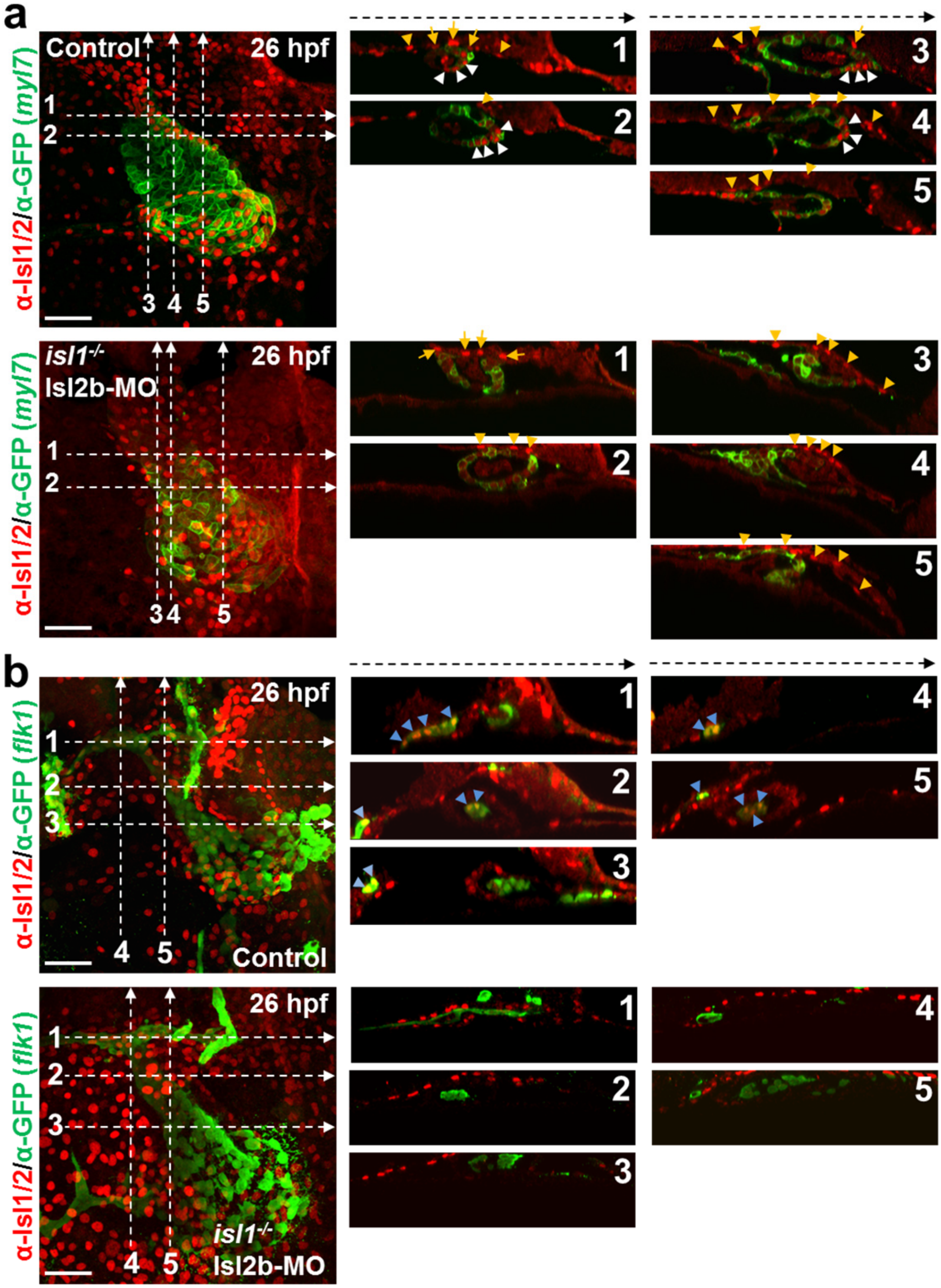
a
Control
26 hpf
α-Isl1/2/α-GFP (myl7)
isl1-/-
Isl2b-MO
b
flk1

Supplementary Figure 2: Distinct patterns of expression of Islet family members in the developing heart.

(a) Confocal images of control and *Isl2b* morpholino-injected *Tg(myl7:EGFP-HsHRAS)*^{s883} *isl1*-/- embryos stained with anti-GFP and anti-Isl1/2 antibodies at 26hpf. Optical sections showing residual Isl2a^{+} cells in the pericardial wall adjacent to the arterial pole (orange arrows) and in endodermal cells on top of the heart tube (orange arrowheads) in *Isl2b* morpholino-injected *isl1-/-* embryos. Isl2b^{+}*myl7*$^{+}$ expressing cells (white arrowheads in wild-type embryos) are lost in *Isl2b* morpholino-injected *isl1-/-* embryos. **(b)** Confocal images of control and *Tg(kdrl:EGFP)*^{s843}*isl1*-/- Isl2b morpholino-injected embryos stained with anti-GFP and anti-Isl1/2 antibodies at 26 hpf. Optical sections showing Isl1^{+}flk1^{+} cells located in the endocardium of the forming ventricle, as well as in the vessels at the arterial pole (blue arrowheads) in wild-type embryos. These cells are not detectable in *isl1*-/- Isl2b-MO embryos, suggesting that they do not express Isl2a. Scale bars, 50 µm.

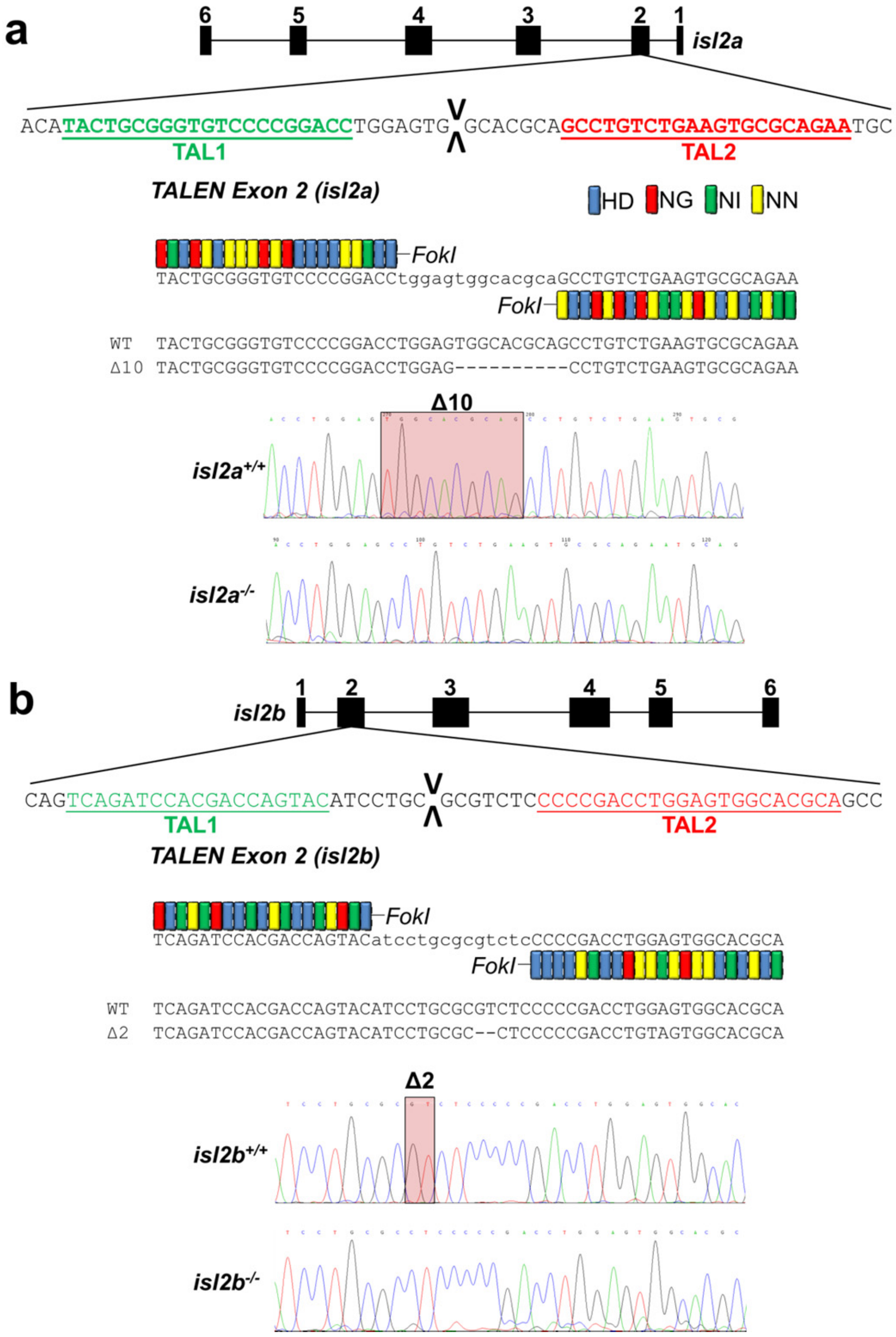

a
6
5
4
3
2
1
isl2a
ACATACTGCGGGTGTCCCCGGACCTGGAGTGGCACGCAGCCTGTCTGAAGTGCGCAGAATGC
TAL1
TAL2
TALEN Exon 2 (isl2a)
HD
NG
NI
NN
FokI
TACTGCGGGTGTCCCCGGACCtggagtggcacgcaGCCTGTCTGAAGTGCGCAGAA
FokI
WT TACTGCGGGTGTCCCCGGACCTGGAGTGGCACGCAGCCTGTCTGAAGTGCGCAGAA
Δ10 TACTGCGGGTGTCCCCGGACCTGGAG----------CCTGTCTGAAGTGCGCAGAA
Δ10
isl2a+/+
isl2a-/-
b
1
2
3
4
5
6
isl2b
CAGTCAGATCCACGACCAGTACATCCTGCGCGTCTCCCCCGACCTGGAGTGGCACGCAGCC
TAL1
TAL2
TALEN Exon 2 (isl2b)
FokI
TCAGATCCACGACCAGTACatcctgcgcgtctcCCCCGACCTGGAGTGGCACGCA
FokI
WT TCAGATCCACGACCAGTACATCCTGCGCGTCTCCCCCGACCTGGAGTGGCACGCA
Δ2 TCAGATCCACGACCAGTACATCCTGCGC--CTCCCCCGACCTGTAGTGGCACGCA
Δ2
isl2b+/+
isl2b-/-

Supplementary Figure 3: Schematic representation of the generation of *isl2a* and *isl2b* mutant lines.

(a-b) TALEN pairs (TAL1 and TAL2) targeting the second exons (TALEN binding sites underlined) of *isl2a* **(a)** and *isl2b* **(b)** were used to induce deletion in the coding regions of these genes. F0 founders were crossed with wild-type fish and F1 heterozygous fish, which carried a 10 bp deletion mutation in the targeted site for *isl2a* and 2 bp deletion for *isl2b* were selected for further studies. The F2 heterozygous progeny were inter-crossed to generate homozygous *isl2a*−/− and *isl2b*−/− embryos.

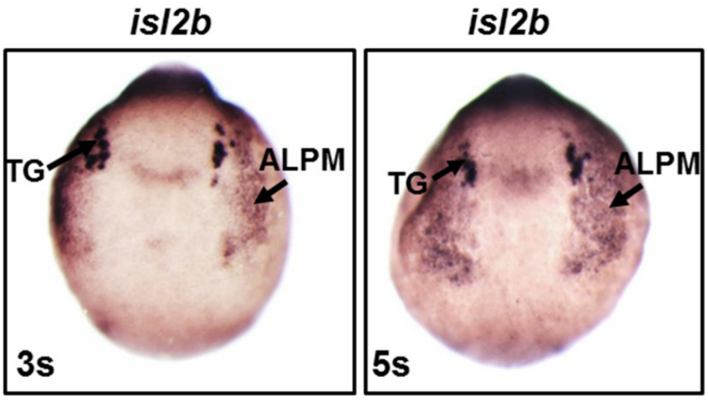

Supplementary Figure 4: Expression of *isl2b* in zebrafish embryos at the 3 and 5 somite stages, detected using *in situ* hybridization. Arrows point to isl2b expressing cells in the ALPM and in the TG (trigeminal placodes).

Mus musculus:	
qGapdh_for	AACTTTGGCATTGTGGAAGG
qGapdh_rev	GGATGCAGGGATGATGTTCT
qIsl1_for	CGGTGCAAGGACAAGAAACG
qIsl1_rev	CCACCATCATGTCTCTCCGG
qMef2c_for	TCCATCAGCCATTTCAACAA
qMef2c_rev	AGTTACAGAGCCGAGGTGGA
qTbx20_for	GCAGCAGAGAACACCATCAA
qTbx20_rev	GTGAGCATCCAGACTCGTCA
qHand2_for	CGGAGAGGCGGAGGCCTTCA
qHand2_rev	CAGGGCCCAGACGTGCTGTG
Danio rerio:	
qEf1alpha_for	CTGGAGGCCAGCTCAAACAT
qEf1alpha_rev	ATCAAGAAGAGTAGTACCGCTAGCATTAC
qIsl1_for	CAAATGGCAGCAGAGCCCAT
qIsl1_rev	GGACGCGGGTTGTTTTCTCA
qIsl1l_for	CTCAGTCCTCGGGTCATTCG
qIsl1l_rev	CTGAAACCAGATGCCCCTCA
qIsl2a_for	GAGTTCTCATTGCGGGACGA
qIsl2a_rev	CTGGCACAGGTTCTGGGATG
qIsl2b_for	TGGCGATAAGACGAATCTGC
qIsl2b_rev	TCCATGGTGGTTGGTAAGCC

Supplementary Table 1: Synthetic oligonucleotides used in the study.
